# 16S rRNA amplicon sequencing of bacterial communities in Solid Waste Leachates (SWL) from Olusosun Dumpsite, Ojota, Lagos State, Nigeria

**DOI:** 10.1128/mra.01244-24

**Published:** 2025-06-30

**Authors:** Adewale K. Ogunyemi, Olanike M. Buraimoh, Bukola C. Ogunyemi, Titilola A. Samuel, Matthew O. Ilori, Olukayode O. Amund

**Affiliations:** 1 Department of Biological Sciences (Microbiology Unit), Trinity University, Lagos, Nigeria; 2 Department of Microbiology, University of Lagos 370949, Lagos, Nigeria; 3 TETFund Centre of Excellence on Biodiversity Conservation and Ecosystem Management (TCEBCEM), University of Lagos, Lagos, Nigeria; 4 Department of Biochemistry, University of Lagos, Lagos, Nigeria; Montana State University, Bozeman, Montana, USA

**Keywords:** 16S rRNA gene sequencing, bacteria community structure, solid waste leachates, acidobacteria, dumpsites, waste control

## Abstract

Here, we use 16S rRNA gene sequencing to identify bacterial community structure of solid waste leachates from Olusosun dumpsite. *Acidobacteria* (14.65 %) was the most abundant phylum with clear affiliations. This was followed by *Planctomycetes* (7. 15 %), *Proteobacteria* (3.28 %), *Chloroflexi* (1.41 %), *Actinobacteria* (0.91 %) and other phyla (0.96 %). Data obtained provides valuable insight into potential strategies for waste management.

## ANNOUNCEMENT

Recent times have seen a rise in the threat of dumpsite contamination due to leachate produced from solid waste disposal, which is strongly influenced by the waste composition, volume of leachate generated, and distance from water bodies (1). The present study aimed to assess the bacterial community structure and diversity in solid waste leachates at Olusosun dumpsite, Ojota, Lagos, Nigeria. The decomposing solid waste was collected from Olusosun dumpsite (coordinates: N 6°29′21.8″; E 003°23′29.3″). Olusosun dumpsite is the most active of all dumpsites in Lagos State (2), the most populous city in sub-Saharan Africa.

The genomic DNA was obtained using the ZR Fungal/Bacterial DNA Kit (Zymo Research, Irvine, CA, USA) following the manufacturer’s instructions. The genomic DNA samples were amplified using universal primers 341F (5′-CCTACGGGNGGCWGCAG) and 785R (5′-. GACTACHVGGGTATCTAATCC) that target the V3–V4 region of the 16S rRNA gene (3). PCR program was run as follows: initial denaturation at 95°C for 3 min, followed by 30 cycles of denaturation at 95°C for 30 s, annealing at 56°C for 30 s, elongation at 72°C for 1 min, and a final elongation step at 72°C for 5 min.

With the use of a MiSeq v3 (600 cycles) Kit, the amplicon was sequenced on Illumina’s MiSeq platform, and about 20 Mb of data (2 × 300 bp long paired-end reads) was generated for each sample. However, Inqaba’s in-house developed data analysis pipeline was employed for BLAST-based data analysis. A standalone Ribopicker v0.4.3 (4) was utilized to remove possible non-rRNA sequences from the raw sequencing reads using the Greengene database (5). FasQC v0.11.9 (6) was used for the quality assessment of the raw amplicon reads. Pre-processing included the use of Trimmomatics v0.39 (7) for the removal of adaptor sequences, low quality (using phred33), and short reads (<100 bp) for both the paired-end files.

**Fig 1:**
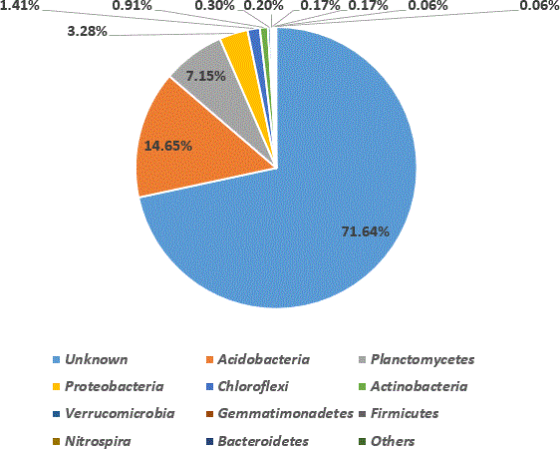
Taxa relative abundance and taxa taxonomic composition of OTUs at the phylum level.

Post-assessed reads were further processed using QIIME2 v2020.6.0 (8) for sequence denoising methods. The denoising method was implemented through the use of DADA2 (QIIME2 plugin:q2-DADA2) (9) with truncating lengths of 284 and 118 for the forward and reversed reads, respectively. Instead of grouping high-quality sequences at a 97 % similarity threshold into operational taxonomic units (OTU), we based on amplicon sequence variants (ASVs), which utilize DNA directly for taxonomic placements instead of clustering of closely related individuals at a 97 % similarity threshold. ASVs were implemented into qiime2 and trained using three databases for comparative taxonomic profiling: SILVA (10), Greengene (5), and Ribosomal Database Project (11). The overall raw sequencing data had 296,086 total reads and an average read length of 173,888 base pairs with 57.86% guanine + cytosine content (Table 1). Fig. 1 depicts taxa relative abundance of major bacterial phyla (>0.05 %). The bacterial communities had an unclear phylum affiliation of 71.64 % abundance. Other bacterial phyla with an abundance ≥ 0.05 % included *Acidobacteria* (14.65 %), *Planctomycetes* (7.15 %), *Proteobacteria* (3.28 %), *Chloroflexi* (1.41 %), *Actinobacteria* (0.91 %), *Verrucomicrobia* (0.30 %), *Gemmatimonadetes* (0.20 %), *Firmicutes* (0.17 %), *Nitrospira* (0.17 %), *Bacteroidetes* (0.06 %), and others (0.06 %) (Fig. 1).

**TABLE 1:** Solid waste leachate amplicon collection features of Olusosun dumpsite

Parameter	Value
Total reads (bp)	296,086
Average read length (bp)	173,888
G + C (%)	57.86
Coverage	68.0031
Size of homopolymer	3
OTU length (bp)	469
OTU total count	145
Inverse Simpson index	0.989
Shannon’s evenness average	6.638
Chao1 richness estimator	256.272
Abundance-based coverage estimator	258.781
Good coverage	0.441
Simpson	0.989

## Data Availability

The targeted locus study project and SRA have been deposited at GenBank with accession numbers KIVW00000000 and SRS22874557, respectively. The BioProject and BioSample accession numbers PRJNA811382 and SAMN28933881 were both obtained from the same raw amplicon sequences.
